# Detection of *Toxoplasma gondii* in artisanal salted meat products sold in street markets of the Ilhéus-Itabuna microregion

**DOI:** 10.1590/S1984-29612024016

**Published:** 2024-03-18

**Authors:** Luane Etienne Barreto, Larissa Araújo Macena, Dhayla Tarine Oliveira de Braga, Nicolli Souza Silva, Brunno Cardoso da Silveira, Daniele de Santana Rocha, George Rêgo Albuquerque

**Affiliations:** 1 Programa de Pós-graduação em Ciência Animal, Departamento de Ciências Agrárias e Ambientais, Universidade Estadual de Santa Cruz - UESC, Ilhéus, BA, Brasil; 2 Departamento de Ciências Agrárias e Ambientais, Universidade Estadual de Santa Cruz - UESC, Ilhéus, BA, Brasil

**Keywords:** Möhr’s Method, artisanal products, serology, PCR, Toxoplasmosis, Método de Möhr, produtos artesanais, sorologia, PCR, Toxoplasmose

## Abstract

This study aimed to detect *Toxoplasma gondii* in artisanal salted meat products sold in street markets in the Ilhéus-Itabuna microregion and to assess the salt concentration used in their preparation and its influence on the parasite’s viability. A total of 125 samples of various artisanal meat products sold in street markets located in the Ilhéus-Itabuna microregion were collected during 2021. Serological analysis using indirect hemagglutination (HAI) and molecular analysis (PCR) were performed on these samples to detect the presence of the parasite. Möhr’s method was utilized to determine the sodium chloride concentration in the samples. Of all samples, 21 were subjected to a bioassay in albino mice to verify the viability of possible tissue cysts. Among the 125 meat products, 10 (8%) tested positive in the serological analysis including four cured pork sausages, five beef sun-dried meats, and one mixed fresh sausage (pork and chicken). None of 125 samples tested positive in the molecular analysis. On bioassay, all mice tested negative for the presence of the parasite. The NaCl concentration in the positive samples ranged from 2.9% to 8%. The results demonstrated that the salt concentration in the collected samples was sufficient to inactivate the parasite *T. gondii*.

## Resumo

Este estudo teve como objetivo detectar *Toxoplasma gondii* em produtos cárneos salgados artesanais, comercializados em feiras livres da microrregião Ilhéus-Itabuna, e avaliar a concentração do sal utilizado no preparo e sua influência na viabilidade do parasito. Foram coletadas 125 amostras de diversos produtos cárneos artesanais, vendidos em feiras livres localizadas na microrregião Ilhéus-Itabuna, durante o ano de 2021. Nessas amostras, a fim de detectar a presença do parasito, foram realizadas análises sorológicas de hemaglutinação indireta (HAI) e molecular (PCR). Para a determinação da concentração de cloreto de sódio presente nas amostras, foi realizado o Método de Möhr. Do total, 21 amostras foram destinadas ao bioensaio em camundongos albinos, na intenção de verificar a viabilidade de possíveis cistos teciduais presentes. Dos 125 produtos cárneos, 10 (8%) foram positivos na análise sorológica, sendo quatro linguiças curadas suína, cinco carnes de sol bovina e uma linguiça frescal mista (suíno e frango). Nenhuma amostra foi positiva na análise molecular. No bioensaio, todos os camundongos testaram negativo para a presença do parasito. Das amostras positivas na sorologia, as concentrações de NaCl variaram de 2,9% a 8%. Os resultados evidenciaram que a concentração de sal das amostras coletadas foi suficiente para inativar o parasito *Toxoplasma gondii*.

## Introduction

*Toxoplasma gondii* is an obligate intracellular protozoan. The definitive hosts of this parasite are domestic and wild felids, while other warm-blooded animals, including humans, act as intermediate hosts. Infection can occur by one of three parasite forms, namely: oocysts shed in the feces of definitive hosts, which become infectious after sporulation in the environment; Tachyzoites, which the most common transmission is congenital; and bradyzoites contained in tissue cysts (Dubey et al., 1998).

Ingestion of tissue cysts is a common form of parasite infection, either through the carnivorous behavior of animals or habit of humans to consume raw or undercooked meat (Lundén & Uggla, 1992). Tissue cysts are relatively resistant to gastric digestion and temperature changes, remaining infectious when refrigerated (1-4°C) for up to three weeks and for over a week at freezing temperatures (between -1 and -8 °C). However, temperatures below -12 °C for 24 hours or temperatures above 67 °C with uniform heating are capable of inactivating *Toxoplasma gondii* cysts (Tenter et al., 2000). Alves et al. (2020) demonstrated that cysts can remain viable in vacuum-packed products for up to 14 days when stored at temperatures of 0 °C ± 1 °C and become inactivated on the 21st day under the same conditions.

Identification of *T. gondii* in animal tissues is common in Brazil (Dubey et al., 2012). In the state of Bahia, several studies of domestic animals, such as chickens (Dubey et al., 2008; Rocha et al., 2018), cattle (Spagnol et al., 2009), pigs (Bezerra et al., 2012) and sheep (Guimarães et al., 2013; Maciel et al., 2014) have revealed a high prevalence of anti-*T. gondii* antibodies resulting from natural infection. Additionally, the parasite has been isolated from tissues and detected molecularly (Dubey et al., 2008; Bezerra et al., 2012; Guimarães et al., 2013; Maciel et al., 2014, Rocha et al., 2018).

Meat products, such as cured, aged, salted and processed meats, can also influence the occurrence of human toxoplasmosis. Salting, curing, and using preservatives in meat products have proven useful in rendering *T. gondii* tissue cysts unviable, however, there are no studies reporting the prevalence and viability of *Toxoplasma gondii* in meat products in Bahia and due to the lack of current legislation and the diversification of techniques used in the production of these artisanal meat products, efficient infection control has not been achieved in many cases (Lundén & Uggla, 1992; Pott et al., 2013; Alves et al., 2019).

Previous research emphasizes the importance of salt concentration and maturation time of meat products before sale, with a minimum recommended concentration of 3% and at least three days of maturation (Jamra et al., 1991). Moreover, in accordance with Navarro et al. (1992), a minimum concentration of 2% with at least two days of maturation is required to inactivate the tissue cysts of *T. gondii.*

None of the studies available in the literature conducted on meat products (Mendonça et al., 2004; Menucci et al., 2010; Marciano et al., 2018; Langoni et al., 2021) assessed the salt concentration and its potential correlation with the inactivation of tissue cysts of *T. gondii*. However, two of them used this hypothesis to justify the false-negative results in experimentally infected animals (Mendonça et al., 2004; Marciano et al., 2018).

In this regard and considering the potential health risk to consumers, this study aimed to detect *Toxoplasma gondii* in artisanal salted meat products sold in street markets in the Ilhéus-Itabuna microregion and to assess the salt concentration used in their preparation and its influence on the parasite’s viability.

## Materials and Methods

### Sample collection

To execute this observational cross-sectional epidemiological study, during 2021, 200g from each of the 125 samples of artisanal meat products were collected using convenience sampling. These samples were obtained from a variety of salted meat products sold in street markets in the cities of Ilhéus (Lat: 14°47'50"S, Long: 39°2'8"W), Itabuna (Lat: 14°47'21"S, Long: 39°16'40"W), Itajuípe (Lat: 14°41'7"S, Long: 39°21'52"W), Uruçuca (Lat: 14°35'12"S, Long: 39°17'29"W), Buerarema (Lat: 14°56'50"S, Long: 39°18'16"W), and Floresta Azul (Lat: 14°50'52"S, Long: 39°39'23"W), Bahia, Brazil (Table 1). After collection, each sample was individually placed in plastic bags and labeled with a corresponding number. During sampling, information about the products was obtained from the vendors, including the date of salting and the amount of salt they used for curing artisanal meat products. This information was recorded for each sample to establish a correlation between the salting time and possible observed characteristics or results obtained in subsequent analyses.

**Table 1:** Artisanal salted meat products and their quantities collected in street markets of the Ilhéus-Itabuna microregion.

**City**	**Sun-dried beef**	**Sun-dried pork**	**Cured pork sausage**	**Fresh mixed sausage**	**Fresh chicken sausage**	**TOTAL**
**Ilheus**	12	-	15	1	1	29
**Itabuna**	10	5	9	-	-	24
**Itajuipe**	12	5	2	-	-	19
**Uruçuca**	10	4	-	-	-	14
**Buerarema**	18	3	-	-	-	21
**Floresta Azul**	14	3	1	-	-	18
**TOTAL**	**76**	**20**	**27**	**1**	**1**	**125**

### Serological detection of anti-*Toxoplasma gondii* antibodies

To detect anti-*T. gondii* antibodies in the collected meat products, the tissues were homogenized, and 1 g of each 125 sample was taken for exudate extraction. Subsequently, the samples were macerated with 4 mL of PBS (phosphate buffered saline) solution and centrifuged at 2000 g for 10 minutes at 4 °C. The serum obtained from this process was transferred to a 2 mL microtube and used for the serological analysis of the samples. In mice, to detect anti-*T. gondii* antibodies, blood collection was performed via submandibular vein (Bogdanske et al., 2010) moments before the euthanasia of each of the 126 inoculated mice. Immediately after collection, the blood was centrifuged and the obtained serum was used for serological analysis. The technique used for the detection of anti-*T. gondii* IgG antibodies was indirect hemagglutination (HAI), using the Toxotest HAI® Kit - Wiener Lab, following the protocol recommended by the manufacturer, considering a cutoff point of 1:16 (Beltrame et al., 2012; Rocha et al., 2018).

### Peptide digestion and biological assay

Fifty grams from all samples with positive serology (10), along with 11 negative samples randomly chosen according to availability, were selected for peptide digestion and biological assay following the protocol of Dubey (1998), totaling 21 samples. For the biological assay, six albino mice (Mus musculus) were used per digested sample, totaling 126 mice. Three mice received subcutaneous inoculation of 1 mL, while the other three received intraperitoneal inoculation of 1.5 mL for two consecutive days with a 24-hour interval between the two inoculations. During the experiment, the mice were fed with specific commercial feed for the species and had ad libitum access to water. They were observed daily for approximately 6 weeks. Mice showing any symptoms of toxoplasmosis underwent blood collection, were subsequently euthanized by CO2 chamber and necropsied to collect organs, including the liver, spleen, heart and brain. Mice without clinical changes were euthanized at 42 days after inoculation (dpi), and the same procedures described above were performed.

### DNA extraction and molecular detection of *Toxoplasma gondii*

All 125 samples of meat products collected and all the collected organs from 126 necropsied mice (totaling 504 organs) were forwarded for DNA extraction. For this, 100 mg of macerated tissue in liquid nitrogen was weighed, and protocol #3 from the Easy-DNA® commercial kit (Invitrogen) was used. After the entire procedure, the extracted DNA was stored in 2 ml microtubes at -20°C until the PCR (Polymerase Chain Reaction) was performed. The DNA amplification of *T. gondii* was performed using the primers Tox4 (CGCTGCAGGGAGGAAGACGAAAGTTG) and Tox5 (CGCTGCAGACACAGTGCATCTGGATT), resulting in a 529 bp fragment (GeneBank N0AFI46527) of *T. gondii* DNA. The PCR was carried out with the extracted DNA (1.5 µl), 16.3 µl ultrapure H_2_O, along with a mixture of each primer (1 µl of forward primer and 1 µl of reverse primer), 1 µl of dNTPs® (Invitrogen), 2.5 µl of Tris-HCl (pH 9.0), 1.5 µl of MgCl2, and 0.2 µl of Taq DNA polymerase® (Invitrogen), resulting in a total volume of 25 µl (23.5 µl of the mix + 1.5 µl of the DNA sample). The reaction was performed in 35 cycles in accordance with the standard protocol, in the following conditions: 5 minutes at 94 °C for initial denaturation in a single cycle, followed by 35 cycles of one minute at 94 °C for denaturation, one minute at 60 °C for annealing, and one minute at 72 °C for Taq DNA polymerase extension, followed by a final extension of 7 minutes at 72 °C. The products of each PCR were subjected to 2% agarose gel electrophoresis, stained with ethidium bromide, and documented by photography. DNA from *T. gondii* tachyzoites was used as positive control and ultrapure water was used as negative control for PCR.

### Detection of sodium chloride levels

Sodium chloride (NaCl) concentration in the collected samples was determined using the titration technique from protocol 5.6 described in the Official Methods Manual for Analysis of Animal Origin Foods (Brasil, 2022). The technique (Möhr’s method) is based on the principle that salt is extracted from the sample with water. After protein precipitation, the chloride concentration is determined by titrating an aliquot of the solution with standardized silver nitrate and calculated using Formula 1 for sodium chloride. The assay was conducted in duplicate for each sample analyzed, and the average of the duplicates was the resulting concentration, expressed in grams of NaCl per 100 g of meat (g/100g):


$$\text{NaCl, in g/100g}\;\frac{\left(V-Vv\right)\;.\;\;f\;.\;\;0.1\;.\;\;58.45\;.\;\;250\;.\;\;100}{m\;.\;\;A\;.\;\;1,000}\;\;$$ (1)

## Results

**Figure 1:**
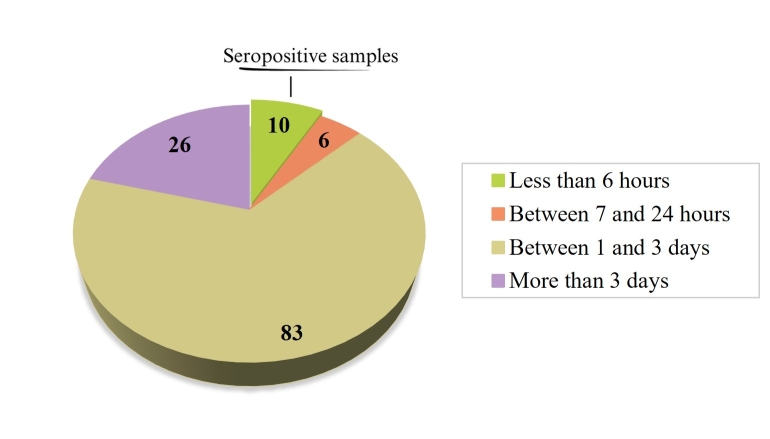
Maturation time of artisanal salted meat products collected in street markets of the Ilhéus-Itabuna microregion.

A total of 125 samples of artisanal meat products sold in street markets of the Ilhéus-Itabuna microregion were gathered (Table 1). None of the producers could precisely indicate the amount of salt added during the product's preparation, describing it as an intuitive process. However, the curing time for all samples was provided (Figure 1).

In the serological analysis to identify anti-*T. gondii* antibodies in the collected meat products, 8% (10/125) tested positive. Among the positive samples, four were cured pork sausages, five were sun-dried beef, and one was a mixed fresh sausage made from pork and chicken meat. All the positive products originated from Ilhéus and had been prepared in the early morning of the collection day, with a maximum product maturation time of 6 hours. In this region, the seropositive rate was 34.5% (10/29); meanwhile, in other regions (Itabuna, Itajuípe, Uruçuca, Buerarema, Floresta Azul) where meat products were collected, the seropositivity was 0%. However, when molecular analysis (PCR) was conducted on the 125 samples, none of them tested positive for the presence of *T. gondii*.

Of the 125 collected samples, 21 were selected for the bioassay, with salt concentrations ranging from 2.8% to 17.6% (per 100g). Among these samples, 10 tested positive in serology and 11 tested negative. Of the 126 inoculated mice, 6 were euthanized before the 42nd day due to clinical alterations, while the remaining mice were euthanized on the 42nd day post-inoculation (dpi). All mice tested negative for the presence of the parasite in both serological and molecular analyses.

The results from salt concentration detection obtained through the Möhr method ranged from 2.2% to 17.7% per 100 grams of meat product. The salt concentration in the seropositive samples ranged from 2.9% to 8%. The average values for each city and their respective products are presented in Table 2.

**Table 2:** Mean salt concentration of meat products collected in street markets of the Ilhéus-Itabuna microregion.

**City**	**Mean [] Salt ± σ/√n (N)**	**Mean [] Beef Sun-Dried Meat ± σ/√n (N)**	**Mean [] Pork Sun-Dried Meat ± σ/√n (N)**	**Mean [] Cured Pork Sausage ± σ/√n (N)**
**Ilheus**	5.32 ± 0.76 (29)	6.25 ± 0.55 (12)	-	4.65 ± 0.3 (15)
**Itabuna**	7.43 ± 0.26 (24)	6.83 ± 0.34 (10)	13.30 ± 3.49 (5)	4.83 ± 0.35 (9)
**Itajuipe**	5.76 ± 0.84 (19)	5.98 ± 0.96 (12)	6.16 ± 1.7 (5)	3.40 ± 1.2 (2)
**Uruçuca**	7.40 ± 1.02 (14)	6.99 ± 1 (10)	8.43 ± 2.87 (4)	-
**Buerarema**	7.67 ± 0.87 (21)	7.39 ± 1.2 (18)	9.33 ± 0.35 (3)	**-**
**Floresta Azul**	5.78 ± 0.54 (18)	5.09 ± 0.79 (14)	9.40 ± 0.44 (3)	**-**

[]: Concentration; σ/√n: Standard error; (N): Total samples.

## Discussion

In this study, among the 10 meat products that tested positive in serology, five were sun-dried beef. This finding supports the indications of Menucci et al. (2010), who highlighted the importance of monitoring sun-dried meat for toxoplasmosis since this type of meat is often prepared without official identity and quality standards and sometimes under inadequate sanitary conditions. Sun-dried meat is a traditional product deeply rooted in Brazil’s northeastern culture and widely consumed in this region (Gurgel et al., 2014). Due to the salting process, it is believed to be a safe product.

All seropositive samples (four cured pork sausages, five sun-dried beef, and one mixed fresh sausage made from pork and chicken meat) found in this study were from the municipality of Ilhéus and had less than 6 hours of maturation, while the other products collected had a minimum maturation time of 10 hours. Previous studies conducted in this region have identified that the animals widely used to produce meat derivatives - pigs (Bezerra et al., 2012), cattle (Spagnol et al., 2009), and free-range chickens (Rocha et al., 2018) - tested positive for *T. gondii* through serological analysis. Additionally, these studies revealed the presence of pathogenic strains when inoculated in mice (Bezerra et al., 2012; Rocha et al., 2018).

Although 10 meat products tested positive in the serological analysis in this study, none of the 125 products collected tested positive in the molecular analysis. Marciano et al. (2018) reported the possibility of salt interference in meat exudates. False-negative results were identified in the serological analysis (ELISA) of sun-dried meat from cattle experimentally infected with *T. gondii*, thus highlighting the importance of salt content when conducting serological analyses of meat products. Additionally, it is essential to note that cysts are not homogeneously distributed and may not be present in the collected fragment or the sample used for bioassay and PCR analysis (Mecca et al., 2011; Marciano et al., 2018).

Although the salt concentration in the serologically positive meat products ranged from 2.9% to 8% in this study, the relatively short maturation time must be considered. As observed from the information during the collection process, the interval between the salting process and analysis was only a few hours. Therefore, the salt had insufficient time to influence the exudate of the samples, considering the minimum salting time required for the inactivation of the parasite, as demonstrated by Jamra et al. (1991) and Navarro et al. (1992).

Regarding the biological assay conducted in this study, the results are in accordance with previous research (Warnekulasuriya et al., 1998; Mendonça et al., 2004; Langoni et al., 2021). None of the 70 samples of pork sausage collected by Mendonça et al. (2004) infected the bioassay mice, but 33 samples tested positive in the molecular analysis (PCR). Mendonça et al. (2004) did not test the salt concentration of the samples, but the authors mentioned that the high salt concentration in the samples may have rendered the tissue cysts unviable for the bioassay.

## Conclusion

This study demonstrated that all seropositive samples were from Ilhéus (10/29) and had a maturation time of less than 6 hours, with a salt concentration ranging from 2.9% to 8% (g/100g). This suggests that despite the short maturation time, the salt content may have been sufficient to inactivate possible cysts in the samples, highlighting the influence of salt concentration and maturation time on the persistence of *Toxoplasma gondii* in meat products.
